# Leaving no one behind: targeting mobile and migrant populations with health interventions for disease elimination—a descriptive systematic review

**DOI:** 10.1186/s12916-022-02365-6

**Published:** 2022-05-09

**Authors:** Molly W Adams, Elizabeth G Sutherland, Erin L Eckert, Khalida Saalim, Richard Reithinger

**Affiliations:** Research Triangle Institute (RTI) International, 701 13th St NW Ste 750, Washington , DC, 20005 USA

**Keywords:** Neglected tropical diseases, Infectious disease, Migrants, Mobile populations, Pastoralists, Treatment, Coverage, Surveillance

## Abstract

**Background:**

Mobile and migrant populations (MMPs) pose a unique challenge to disease elimination campaigns as they are often hard to survey and reach with treatment. While some elimination efforts have had success reaching MMPs, other campaigns are struggling to do so, which may be affecting progress towards disease control and elimination. Therefore, this paper reviews the literature on elimination campaigns targeting MMPs across a selection of elimination diseases—neglected tropical diseases, malaria, trypanosomiasis, polio, smallpox, and rinderpest.

**Methods:**

Through a systematic review process following Preferred Reporting Items for Systematic Reviews and Meta-Analyses (PRISMA) guidelines, a three-person review team identified papers from databases, conference records, and citation searches using inclusion/exclusion criteria. Papers were divided into three key outcome domains during the synthetization process: (1) MMP movement patterns in East Africa including reasons for movement and consequences in terms of health outcomes and healthcare access; (2) MMP contribution to the transmission of disease across all geographies; (3) surveillance methods and treatment interventions used to implement programming in MMPs across all geographies. Experts in the field also provided supplemental information and gray literature to support this review.

**Results:**

The review identified 103 records which were descriptively analyzed using the outcome domains. The results indicate that in East Africa, there are various motivations for migration from economic opportunity to political unrest to natural disasters. Regardless of motivation, mobile lifestyles affect health service access such that MMPs in East Africa report barriers in accessing healthcare and have limited health knowledge. Often lower service delivery to these populations has resulted in higher disease prevalence. A minority of articles suggest MMPs do not pose challenges to reaching disease control and elimination thresholds. Finally, the literature highlighted surveillance methods (e.g., using satellite imagery or mobile phone data to track movement, participatory mapping, snowball sampling) and intervention strategies (e.g., integration with animal health campaigns, cross-border coordination, alternative mass drug administration [MDA] methods) to implement health interventions in MMPs.

**Conclusions:**

Ultimately, the literature reviewed here can inform programmatic decisions as the community attempts to reach these never treated populations.

**Systematic review registration:**

The protocol for this manuscript was registered with the International Prospective Registry of Systematic Reviews (PROSPERO) (No. CRD42021214743),

## Background

Neglected tropical diseases (NTDs) are a diverse group of 20 major disabling conditions, mainly of parasitic and bacterial etiology. They impact over 2.5 billion people worldwide, primarily in rural and poor urban settings, where access to clean water or safe disposal of human waste are major drivers of exposure and risk [1]. If left untreated, NTDs can cause great morbidity, which often results in substantial disability affecting quality of life and productivity of those affected [2]. Five major NTDs (i.e., trachoma, lymphatic filariasis [LF], onchocerciasis, schistosomiasis [SCH], and soil-transmitted helminths [STH]) can be controlled or eliminated through mass drug administration (MDA) of preventive chemotherapy (PC) [3]. Over the past decade, there has been great progress in the control and elimination of these PC-NTDs [4].

While this progress is positive, the NTD community continues to face challenges in reaching control and elimination targets. Population growth and mobility have increasingly been observed as a hindrance to this progress [5]. First, if programmatic surveys include randomly selected enumeration units with a high proportion of mobile and migrant populations (MMPs) affected by NTDs, such surveys may over-estimate the intensity of autochthonous transmission (i.e., since MMPs could have been infected outside of survey units, and/or certain populations are historically known to be at greater risk of NTD infection). Second, NTDs could be re-established in areas where these have been eliminated (e.g., if MMPs affected by NTDs migrate to an area where MDAs have stopped). Third, NTD interventions often face challenges in targeting MMPs, due to their limited geographic access to health facilities and NTD testing, poor existing health service quality delivered to these populations, interruptions in treatment supply, high out-of-pocket payment and travel costs, and low awareness of and alternative beliefs on NTDs and treatment [6, 7]. In other circumstances, MMPs may be excluded from health programming due to cultural and language barriers, as well as stigma, possible illegal status, resulting in a fear of deportation [8].

The East African region is characterized by high migration rates and has experienced various migration flows and patterns [9]. MMPs in East Africa typically include populations who (i) seek labor and follow agropastoral traditions (e.g., livestock herders, seasonal laborers, forest workers, miners); (ii) are involved in or are the result of insecurity and conflict (i.e., military personnel, armed militia, internally displaced people [IDPs], and refugees); or (iii) are the result of humanitarian emergencies (i.e., IDPs, and refugees) [10, 11]. In 2019, East Africa alone hosted 7.7 million international migrants along with 3.6 million refugees and asylum seekers [12, 13].

We conducted a systematic review to better understand the movement patterns of MMPs, their effect on disease transmission dynamics, and the subsequent consequences on programmatic disease elimination efforts (such as NTD campaigns) in endemic areas. The intended objectives of this review were (1) to establish an understanding of motivation for and consequences of MMP movement patterns; (2) to understand how these MMPs affect the transmission of infectious diseases; (3) to identify tools being employed to monitor MMP movement patterns and to develop more effective approaches and strategies to reach MMPs with health interventions. For the first objective, we limited our search to East Africa as this region is of specific interest to the global infectious disease community: there is substantial known cross-border movement, specifically of the Turkana and Maasai, that impact disease control and elimination efforts [14, 15]. For the second and third objectives, we broadened our review to include other geographies. This broader scope was intentional to ensure we captured the breadth of epidemiological information on disease prevalence in MMPs and to include all programmatic approaches and strategies used to reach MMPs with health interventions.

To better understand lessons learned that are applicable to NTDs, we also explored other formerly prevalent and successfully eliminated—or nearly eliminated—viral pathogens (i.e., rinderpest, smallpox, polio) as well as current parasitic diseases (i.e., trypanosomiasis, malaria). We examined the application of specific successful and unsuccessful interventions used to reach MMPs with disease programming for continued progress towards disease elimination. Here forth all diseases included in the review will be referred to as “focus diseases.”

## Methods

### Definition of MMPs

We define MMPs as groups of people who do not reside in one location for long periods of time but rather travel sporadically or seasonally due to their occupation, lifestyle, political unrest, or environmental hazards [11].

For the purpose of this review, we excluded stationary populations (such as refugees, migrants, or internally displaced persons who reside in camps). This distinction was made to focus on mobile populations (e.g., migrant workers, nomadic pastoralists) who may be hard to capture in a census or other types of sampling frames.

### Eligibility criteria

The descriptive systematic review included articles published from 2000 to 2021 written in English, Spanish, French, German, Portuguese, and Italian. We included articles reporting on MMP movement patterns in East Africa, epidemiology of focus diseases affecting MMPs, and tools and strategies used to monitor MMP movement and target MMPs affected by focus diseases with programmatic interventions. We included both quantitative and qualitative studies; however, we did not include other systematic reviews, literature reviews, and meta-analyses.

We added Kenya, Tanzania, and Uganda as specific search terms because that is where we see an immediate programmatic need to address issues of persistent transmission in a handful of districts that have embarked on last mile efforts to eliminate PC-NTDs. We expect many last mile issues related to PC-NTDs in these countries to reflect other elimination challenges faced elsewhere due to MMP movement.

We excluded papers reporting on general mHealth approaches not tailored to MMPs. Additionally, we excluded papers on travel medicine, disease serology and diagnosis specificities, persons living settled lifestyles, and papers reporting on MMP socio-demographics and anthropology with no relevance to health or movement patterns.

The included studies fell into three groups for synthesis: (1) studies on movement patterns and healthcare seeking practices of MMPs in East Africa; (2) studies reporting on the prevalence of focus diseases in MMPs and MMP impact on disease transmission globally; (3) studies on methodologies used to enumerate MMPs, track their movement, and reach them with treatment and interventions.

The study protocol was registered through the International Prospective Registry of Systematic Reviews (PROSPERO) (No. CRD42021214743).

### Information sources

We developed the review process in alignment with the Problem/Population, Intervention, Comparison, and Outcome (PICO) framework [16]. Through this process, we finalized the search terms and conducted a trial run to ensure the results were as intended.

The systematic literature search utilized five databases: AnthroSource, EBSCO, PubMed, OVID Medline, and Web of Science. We also consulted with 12 experts in the field (see Acknowledgements) to identify published and gray literature that could have been missed through the database search. Using a snowball method, we manually examined the reference lists from systematic reviews and included a subset of articles using the inclusion/exclusion criteria.

Additionally, we carried out a manual systematic search of conference abstracts dating back to 2010. For this search, we reviewed the American Society of Tropical Medicine and Hygiene (ASTMH), Coalition for Operation Research on NTDs (COR-NTD) and the Neglected Tropical Disease Non-Governmental Development Organization Network (NNN) conference records and selected abstracts using the above inclusion/exclusion criteria.

### Search strategy

We systematically searched the five databases using specific search terms (see Table 1). We conducted two searches based on the intended outcomes from the different types of databases. First, we searched Anthrosource and EBSCO for anthropological literature about MMPs in East Africa, their movement patterns, and their customs as they relate to healthcare access and acceptance of health services and interventions. We limited this search to East Africa as we were interested in understanding the movement patterns and healthcare-related practices of MMPs in this area, such as the Turkana and Maasai peoples, their high disease prevalence, frequent mobility, and known challenges to accessing treatment faced by these two groups and others [14, 15].

**Table 1:** Search terms

Dimension	Term	Connector
Problem	neglected tropical disease* OR “neglected tropical disease*”.mp. OR exp Neglected Diseases/ OR TS= (“neglected tropical diseases”) OR trachoma [MeSH Terms] OR trachoma.mp. OR exp Trachoma/ OR TS= (trachoma*) OR lymphatic filariasis OR elephantiasis OR “lymphatic filariasis”.mp. OR Elephantiasis, Filarial/ OR TS= (lymphatic filariasis* OR elephantiasis) OR onchoceriasis [MeSH Terms] OR onchocerciasis.mp. OR exp Onchocerciasis, Ocular/ OR Onchocerciasis OR TS= (onchoceriasis*) OR schistosomiasis [MeSH] OR schistosomiasis.mp. OR Schistosomiasis/ OR TS= (schistosomiasis*) OR “soil transmitted helminths” OR “soil transmitted helminths”.mp. OR exp Helminthiasis/ OR TS= (“soil transmitted helminths”) OR malaria [MeSH] OR malaria.mp. OR exp Malaria/ OR TS= (malaria) OR polio [MeSH] OR (acute poliomyelitis [Mesh Terms]) OR polio.mp. or exp Poliomyelitis/ OR TS= (polio) OR smallpox [MeSH] OR exp Smallpox/ or smallpox.mp. OR TS= (smallpox) OR rinderpest [MeSH] OR exp Rinderpest virus/ or exp Rinderpest/ or rinderpest.mp. OR TS= (rinderpest) OR trypanosomiasis [MeSH] OR trypanosomiasis.mp. or exp Trypanosomiasis/ OR TS= (trypanosomiasis)	AND
Population	migrat* OR migrant* OR (migrat* OR migrant*).mp. OR TS= (migrat* OR migrant*) OR “mobile populations“ OR “mobile populations”.mp. OR TS= “mobile populations” OR transients and migrants [MeSH Term] OR “Transients and Migrants”.mp. OR exp “Transients and Migrants”/ OR TS= transient* OR mobile OR mobile.mp. OR TS= mobile OR pastoralis* OR pastoralis*.mp. OR TS= pastoralis* OR nomad* OR nomad*.mp. OR TS= nomad* OR Turkana OR Turkana.mp. OR TS= Turkana OR Maasai OR Masai OR (Maasai or Masai).mp. OR TS= (Maasai or Masai)	AND
	*Geography specific terms used in Search 1 referenced above* “East Africa” OR Kenya OR Tanzania OR Uganda	AND
Intervention	“Insect Control”[Mesh] OR “insect control”.mp. OR TS = insect control OR "Mass Drug Administration"[Mesh] OR “Mass Drug Administration”.mp. OR TS = “mass drug administration” OR “Mass Vaccination”[Mesh] OR “mass vaccination”.mp. or exp Mass Vaccination/ OR TS = (“mass vaccination”) OR “Sanitation”[Mesh] OR “Hygiene”[Mesh] OR sanitation OR hygiene OR TS = (sanitation OR hygiene)	AND
Outcome	(“public health surveillance” [Mesh]) OR (“sentinel surveillance” [Mesh]) OR exp public health surveillance/ or exp sentinel surveillance/ OR TS= (public health surveillance) OR (sentinel surveillance) OR “Epidemiological Monitoring”[Mesh:NoExp] OR exp Epidemiological Monitoring/ OR TS = “epidemiological monitoring” OR “Geographic Mapping”[Mesh] OR exp geographic mapping/ OR TS= (geographic mapping)	--

For the second search, we did not restrict the geographic focus because information on persistent transmission due to MMPs and lessons learned from targeting MMPs with MDA campaigns can be applied more universally. We harvested results from PubMed, Ovid MEDLINE, and Web of Science on papers discussing disease prevalence in MMPs, tools to monitor and enumerate focus diseases in MMPs, and intervention methods and strategies to provide focus disease programming to MMPs.

**Fig. 1:**
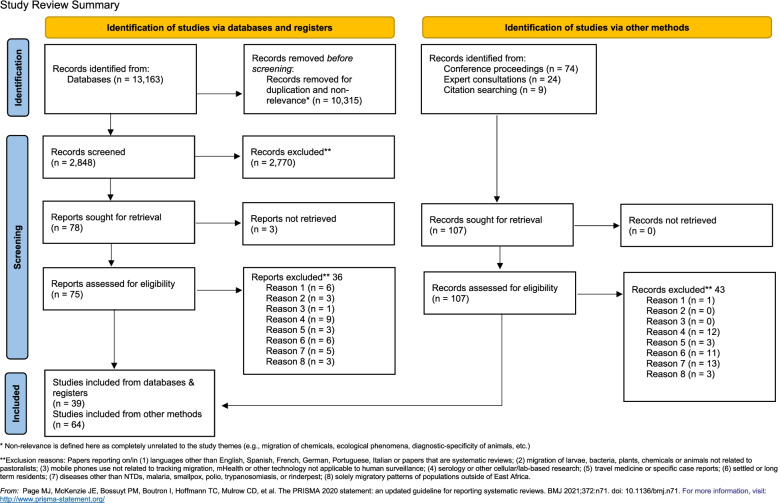
PRISMA flow diagram

From the initial database search using the terms in Table 1, we pulled 13,163 articles. While this result was much larger than originally anticipated, we concluded that limiting the search terms or reducing the number of databases searched could lead to missing relevant methodological articles. Therefore, we conducted a pre-title review phase during which we excluded any articles of irrelevance based on title. This stage removed all articles on animals or plants unrelated to MMPs, reports on diseases not included in our focus diseases, papers on the environment, papers on migration of larvae and migration of chemicals, and cellular-level biology reports. Following this step, 2848 articles moved forward to further review. In this second step, another title review was conducted, as well as a review of the abstract and lastly full text. Applying this phased review process resulted in the inclusion of a total of 39 articles in the review (Fig. 1).

We also systematically searched conference proceedings for relevant conference abstracts. As advanced search boxes were not available, we used the find (control “f”) feature to locate the following search terms in the abstracts: “migrant,” “mobile,” “nomad,” “pastoralist” “transient.” From this search, we identified and reviewed 74 abstracts, of which 34 were ultimately included in the review.

### Selection process

We employed a two-person review team (MA, ES) to engage in every step of the three-stage article review process. The team read every title, abstract, or full text and made an inclusion/exclusion decision based on the criteria. If an article was excluded at the full text stage, we coded it with a number corresponding to the criteria for which it was excluded. Once we completed the reviews, we analyzed the results for discrepancy. We sent any articles with conflicting inclusion/exclusion decisions to a third reviewer (KS) to “tie break.” We repeated this process for the title, abstract, and full text review stages, as well as for the conference abstracts.

### Data collection process

EndNote (Clarivate Analytics, London, UK) was used to organize records and share information among the review team. Records were tagged with key words in EndNote (Table 2) and findings from each paper were synthesized in 3–5 sentence summaries on a shared spreadsheet. As this review included mostly qualitative articles on intervention methodology, we did not find it necessary to confirm data and results from the study authors. No automation tools were used in this process.

**Table 2:** Key work tags for synthesis

	Outcome domain		
	MMP movement patterns and access	Focus diseases in MMPs	Methods for targeting MMPs for health interventions
**Tags**	Kenya, Tanzania, Uganda, [country specific tag], MMPs, Movement patterns, Migrant laborers, Nomadic pastoralists, Maasai, Turkana, Refugees, IDPs, Healthcare access	NTDs, Trachoma, LF, onchocerciasis, SCH, STH, Other NTDs, Malaria, Smallpox, Polio, Zoonotic diseases, Rinderpest, Trypanosomiasis	Alternative MDA, vaccination, cross-border intervention, OneHealth, community access, commodity movement, educational, surveillance, sampling, modeling, mapping, satellite imagery, mobile phone tracking

### Outcome domains

We analyzed the outcomes using three domains, which were aligned with the review’s objectives. First, we synthesized data on MMP movement patterns in East Africa, motivation for moving, seasonal habits, and cultural practices related to healthcare. This outcome domain was descriptive in nature, providing detail on how these populations move, how accepting MMPs are of health interventions, and consequences of movement such as lack of access to treatment. Second, we grouped data based on prevalence results of focus diseases in MMPs and how MMPs contribute to persistent transmission of focus diseases. Rather than focusing on specific prevalence estimates, this outcome domain was included to establish the effect of population movement on disease transmission. For the third outcome domain, we synthesized literature on population enumeration tools and intervention methods used to treat MMPs with mass campaigns for focus diseases. We aimed to collect data on a myriad of existing methodologies used to target these populations with treatment interventions.

### Study risk of bias assessment

Our central motive for engaging three individuals in the review process was to reduce bias in the study selection process. At least two, if not three, independent reviewers assessed every article. The experts consulted came from a variety of backgrounds and health-specific fields. Their recommendations were diverse as a result, suggesting little risk of bias.

### Effect measures

This review was qualitative in nature and therefore assessing effect measures of the outcomes did not apply.

### Synthesis methods

We split final records—studies, gray literature, and conference abstracts—evenly among the three reviewers to determine grouping for synthesis. We tagged each record with key words based on the outcome domains. The synthesis key words can be seen in Table 2. We identified the key words, or thematic codes, prior to the full text paper review and included them in a coding dictionary. All three reviewers used the coding dictionary to ensure consistency in the use of key words and facilitate synthetization. The key words describe geography, focus diseases, methodological approaches, and the main elements of the three study questions. Additionally, we included the key word in the shared database of resources to streamline the thematic analysis process. The results of that thematic analysis are included in the “Results” section.

### Reporting bias assessment

The review was exhaustive in scope as illustrated by the size of the initial literature search pull, minimizing the risk of reporting bias and missing results. We conducted the review systematically with three individuals reviewing the articles and the results. We conducted a snowball sample to ensure any relevant articles missed from the first literature pull were included. Additionally, we conducted another search 4 months after the first pull to capture any articles published during that time period. Therefore, we believe that it is unlikely that the review team missed crucial articles.

### Certainty assessment

We did not conduct a formal certainty assessment as it was not applicable in this review. The objective of the review was not to evaluate the results from quantitative nor quasi-experimental literature. Instead, we aimed to provide an overview of MMP movement patterns in East Africa, the impact of MMPs on disease transmission, and the methods used to enumerate and treat them. It is for this reason we did not attempt to conduct a certainty assessment nor a meta-analysis.

## Results

### General paper characteristics

In final, we identified 103 separate records: 39 articles from the database search; 34 conference abstracts from the conference record review; 22 records identified by key experts, including gray literature, published material, and meeting records; and, lastly, 8 articles from a snowball review of all NTD-related articles’ reference lists and reference lists from systematic reviews of relevance that were captured in the database pull (Fig. 1).

Studies were mostly conducted in Africa (*n* = 45), followed by Asia (*n* = 29) and South America (*n* = 7); one paper reported on MMPs and focus diseases in Australia (*n* = 1). Records range in date from 2000 to 2021, with a median value of 2016 (Table 3). The sample sizes ranged from zero (in the case of editorials or topic reviews), to primary data collection with small numbers (i.e., 6 focus group discussions), to large surveys of tens of thousands of individuals.

**Table 3:** Results from systematic review search

Theme		Characteristics of MMPs and their movement	MMP contribution to disease transmission	Implementing mass treatment campaigns in MMPs
Total		[14, 15, 17–23]	[24–71]	[8, 72–115]
Location	East Africa	[14, 15, 17, 19–23]	[27–32, 34, 35, 67, 68, 70]	[76, 86]
	Other Africa		[33, 37, 45, 46, 48, 56, 58, 63, 69, 116, 117]	[72, 74, 79, 82, 89, 96, 98, 100, 101, 107, 108, 111]
	Asia		[24, 36, 40, 44, 47, 53, 55, 57, 59, 61, 62, 64–66]	[81, 83, 84, 87, 88, 91–94, 99, 102–106, 110, 113]
	Other	[18]	[26, 41–43, 52, 54, 60]	[95]
Disease	NTDs	[15, 23]	[25–32, 34–50]	[72, 81, 89, 90, 97, 101, 105, 109]
	Malaria	[14, 18–22]	[24, 33, 51–65, 116, 118]	[8, 73, 74, 77, 83, 84, 87, 91–94, 99, 102–104, 106, 107, 110, 113]
	Others		[67–69]	[79, 82, 96, 100, 108]

### Study designs and risk of bias

Most of the papers either described generally or presented experimental analysis from new methodologies (*n* = 32) or reported on results from cross-sectional surveys (*n* = 32). The review included studies using descriptive analyses (*n* = 17), mixed method approaches (*n* = 13) and qualitative interventions (*n* = 6). Three papers described longitudinal (*n* = 2) and case control studies (*n* = 1).

Given the descriptive nature of the systematic review, we did not conduct a risk of bias assessment of the included studies.

### Outcome domains

We synthesized results based on the three outcome domains. The first domain included epidemiologic and anthropologic papers on MMP movement patters in East Africa (*n* = 9). We identified two sub-domains: reasons for MMP movement and consequences in terms of health outcomes and health access of movement. The second domain focused on how MMPs contribute to the transmission of disease (*n* = 48). We synthesized cross-sectional, mixed method, qualitative, and descriptive articles to flesh out the literature pertaining to this theme. For the third domain, we examined method studies to provide insight to the final theme discussing methods and interventions to monitor MMP movement and target them with preventive treatment (*n* = 45). We further divided this domain into two sub-domains: tools to monitor and sample MMPs and their movement, and intervention strategies to target MMPs with mass treatment campaigns.

#### Outcome domain 1: MMP movement in East Africa

Overall, the literature search identified nine articles describing East African MMP motivations for moving, attitudes towards healthcare, and access to health services [14, 15, 17–23].

##### Motivation for movement

Our search yielded studies focusing on different types of MMPs—nomadic pastoralists, migrant laborers, IDPs, and refugees—each with various motivations for movement. Common migrant population definitions specify whether movement is cross-border or within single countries, being driven by the demands of livestock (nomadic pastoralists), in pursuit of economic opportunities (migrant laborers), or in response to natural disasters or conflict (IDPs and refugees) [18]. For example, two specific groups of interest, the Maasai and Turkana, move seasonally to access water sources for their cattle [19]. The literature describes their movement as independent of borders, contributing to persistent malaria transmission specifically [20, 22]. Of relevance for NTD epidemiology, motivation for movement is often associated with demographic characteristics that may also be associated with NTD risk. For example, economic migrants are often young and/or single adults, while IDPs or refugees moving in response to conflict or natural disaster are often comprised of family units that include women and children. Furthermore, barriers that women face due to their MMP status can also be exacerbated by their minority gender status [8]. Among pastoralist communities, movement patterns may be different for women and children than for male youth and adults who are moving livestock in certain seasons. Where the demographics of MMP groups and risk for NTDs overlap in endemic receiving or sending areas, programs can identify priority populations for NTD interventions.

##### Consequences of movement

We found three studies discussing the difficulty of reaching MMPs with health services in East Africa [17, 19, 23]. These papers described the issue specifically in the Maasai people in Kenya and Tanzania. Lawson et al. [19] found that the Maasai face barriers to healthcare access due to inadequate service provision in their remote areas. Therefore, when comparing Maasai health to other nomadic groups, the authors found that levels of child malnutrition and disease were very high. Mtuy et al. [23] observed that Maasai seemingly had limited health knowledge as interviews indicated that there is an erroneous belief that trachoma is caused by environmental allergens. An additional four studies illustrated low levels of engagement in healthcare services due to distrust of western medicine or misinformation about health service campaigns. Consequently, studies observed worse health outcomes in the MMPs, such as increased burden of malaria and trachoma [15, 20–22]. In contrast, one study comparing health issues of settled and nomadic Turkana in Kenya reported that the settled Turkana suffered from higher rates of infections like eye infections, colds, coughs, and respiratory infections than the nomadic Turkana [14]. The majority of these studies’ findings were corroborated by our expert consultations, specifically about the types of MMP movement (e.g., cross border, seasonal, labor-specific travelers) in East Africa and how this mobility has affected focus disease service delivery like mass drug and vaccination campaigns in the region.

The studies describing East African MMPs, as well as additional records from domains two and three, also highlighted variables of population movement—such as timing, duration of movement, demographics of those moving, and border crossing—that affect how MMPs utilize healthcare services. Timing can affect health outcomes by exposing MMPs to environmental risks or impeding physical access to services (e.g., by increasing remoteness or by the degradation in roads, tracks or trails, or localized flooding in the wet season) [17, 23, 24, 119, 120]. Duration of migration can similarly affect exposure, disease risk, or access to services that are only available on a local or regional basis [20, 25–27, 72]. Demographics are important when considering diseases that have outsize effects based on age or gender, and border crossing exposes migrants to the policies and practices of different government health care systems which might require international coordination and collaboration to prevent gaps in coverage [18, 28–32, 73, 121]. Demographics are also an important consideration when migration flows are of large size and/or are unpredictable (such as when movement is dependent upon climate conditions, conflict, or natural disaster) as this can lead to situations where the health care infrastructure is not prepared to absorb or respond to the numbers requiring intervention [25, 33, 74, 75, 122].

#### Outcome domain 2: MMP contribution to disease transmission

Altogether, 50 papers directly contributed to this outcome domain. Of these, 25 discussed NTDs [25–32, 34–50] and 20 discussed malaria only [24, 33, 51–66, 116, 118]; five papers focused on a mix of health outcomes, including trypanosomiasis and polio [67–71]. Many of the variables affecting population movement identified in the first theme carried over into this question as specific factors affecting the transmission of focus diseases by and within MMP populations.

Many reviewed studies reported MMPs being missing or underrepresented in service delivery programs [28, 29, 31, 34, 35, 37–39, 41, 51, 54, 56, 57, 60, 67, 69–71]. This underserved status, it is argued, is important to resolve to address both equity concerns and modeling evidence indicating that focus disease control and elimination efforts cannot be successful without adequate intervention coverage among MMPs. One study comparing polio vaccination rates of settled and nomadic populations in western Kenya found significantly different vaccination rates in settled versus nomadic children under 5 years of age (i.e., 85% vs 28%) [67]. This discrepancy in coverage is further concerning when noting that mobility may increase transmission dynamics and affect resource allocation at and between both sending (the location of origin for migrant flows) and receiving (destination locations) areas that may have varying endemicity statuses. In a study on the potential risk of re-infection of LF in Togo, a country with documented LF elimination, Dorenkoo et al. surveyed multiple MMP groups from neighboring countries with known travel routes through Togo [37]. They concluded that the nomadic Peuhls, with an LF prevalence rate of 11.9%, pose a risk of potentially reintroducing LF into Togo. Furthermore, a study on imported malaria cases in Suriname found that between 2006 and 2015, imported cases of malaria increased from 6.8 to 79.5% due to high migration rates of migrant laborers [54]. Most Surinamese cases (94%) remained within the migrant community, but cross-border movement of migrant laborers continued to pose risk of reintroduction to the local community. As highlighted in much of the reviewed literature, reintroduction and continued transmission of focus diseases due to MMP movement poses challenges to achieving their control and elimination.

Some articles presented data that suggest MMPs are not a barrier to control or elimination of focus diseases. For example, in Senegal, there was a concern that migration during rainy season would increase malaria prevalence. However, a study by Thwing et al. [58] found that parasite prevalence was low (0.5%) among the nomad population, suggesting they posed very little risk of causing transmission during travel. Lindblade et al. [42] came to a similar conclusion when determining the prevalence of onchocerciasis in Guatemala and the risk that coffee harvesting migrant workers pose to recurrent transmission. The authors tested migrant workers for the presence of IgG4 antibodies to a recombinant Onchocerca volvulus antigen and found a sero-prevalence rate of 0.6%, concluding that these workers play an insignificant role in onchocerciasis transmission. While it is important to note settings where MMPs seemingly do not contribute majorly to disease transmission, such examples seem to be rarer in the literature. The review did not identify commonalities in disease type, geography, or epidemiology between the studies that suggest MMPs are posing a challenge to control and elimination of focus diseases.

If MMPs reside in areas that do receive services for focus diseases, service and intervention coverage can still be an issue. The literature documents known instances of low coverage or inaccurate coverage reporting due to MMPs’ mobility. In attempting to collect baseline trachoma prevalence in a nomadic community in Australia, Lansingh et al. [41] conducted trachoma examinations four times over the course of 13 months. They reported an overall examination rate of 75%; however, the examination rate for any one visit was between 15 and 53%. Additionally, only two of the 485 participants examined were examined during all four examinations.

Lack of access to services can be mediated by differences in MMPs’ knowledge, attitudes, and practices (KAP) that might place them at greater risk for infection and disease (e.g., in terms of exposure, preventive behaviors) and/or affect health seeking behaviors in such a way that treatment through the health system is less likely [24, 36, 44, 58, 60–62, 64–68, 116, 118]. For example, in many surveys, MMPs are less likely to recognize symptoms of disease, understand how diseases are spread, and have access to safe water, adequate sanitation, and proper hygiene education. In one study on the prevalence of SCH in a migrant community in China, KAP survey results suggested that only 43.9% of migrants sampled had knowledge of SCH control measures [36]. Another study compared polio vaccination knowledge of settled persons and nomadic pastoralists in Kenya, with 15% of nomadic mothers reportedly knowing when a child was supposed to be receiving a vaccine compared to 67% of settled mothers [67]. This discrepancy in KAP can affect coverage among these populations; in addition, language barriers may exacerbate access to and understanding of health education and health services.

As noted for the first outcome domain, other characteristics of MMPs also mediate their risks or affect access to local health education or services. These include socio-economic demographics (including age and gender), duration of stay (e.g., overtime migrants may assimilate with residential populations), and settlement patterns (e.g., MMPs may be integrated in established communities vs transient camps) which all bring with them unique risk factors as well as varying access to health education and services. In short, it is impossible to think of MMPs as a monolith with a single effect on transmission dynamics.

#### Outcome domain 3: Implementing mass treatment campaigns in MMPs

Forty-four articles were identified that directly contributed to our understanding of this third study question [8, 72–114]. All articles were designing, describing, or testing an approach to better monitor MMPs and describe their contribution to ongoing disease transmission.

##### Tools to monitor, map, and sample MMPs

One of the major stumbling blocks to understanding disease dynamics in MMPs is the difficulty in enumerating and surveying these groups. Commonly used means of constructing sampling frames, such as censuses or household enumeration, are not designed to capture and sample MMPs. We found twelve articles describing alternative methods to enumerating MMPs (see Table 4). Sampling strategies include the use of geospatial data to identify movements, tents, or settlements [86, 98, 101, 104, 111]. Study results suggest these alternative sampling frames can be comparable to standard methods. For example, a study in Cameroon on mapping nomadic pastoralist movement found that more than 75% of cattle camps identified as probable through satellite imagery were found to be camps upon manual, on-the-ground confirmation [101]. In addition to satellite imagery, tracking mobile phones or use of mobile phone apps has also been used to map the movement patterns of migrants and determine the length of stay and locations along a migration route [72, 77, 95, 107]. For example, Tomkins et al. [107] used mobile phone data to analyze Senegalese migration patterns and how they may affect malaria transmission. Their study found that 60% of people have recurring trips to the same location and most visits include an overnight stay which increases the risk of malaria infection. Albeit cell phone data can provide accurate information to determine travel routes, it is limited to those MMPs with phones and areas with good cellular reception, possibly excluding low-income populations and those in very rural settings. Additionally, cross-border migration may not be tracked if MMPs do not access different cell phone service providers networks operating on the other side of the border. Snowball or respondent-driven sampling has also been used to survey MMPs with success [102, 110]. Using focus group discussions and key informant interviews, Smith et al. [102] found that 54% of Nepalese malaria cases were imported from India due to work travel. Imported malaria cases were observed more in males (85%) than females and suggested that longer trips were more predictive of malaria infections. This study exemplifies that alternative sampling methods can be successfully used to enumerate MMPs when traditional methods cannot be applied.

**Table 4:** Summary of enumeration methods to sample MMPs for disease monitoring and surveillance

Study	Country	Target MMP group	Disease sampled	Type of paper/study	Method	Results
Garcia et al., 2014 [77]	Global	MMPs	Malaria	Mixed methods	Analyzed (micro)census, survey, and cellphone-based human population movement (HPM) data to map the connectivity of country and subnational AU through population movement. Combined these data with malaria transmission maps and global population dataset to identify hot spots of transmission and imported infection.	Certain regions and countries are more strongly connected because of high levels of HPM. Maps can be used to inform design of malaria elimination strategies by identifying regions that are less connected by HPM and therefore at less risk of re-transmission.
Giada et al., 2003 [86]	Tanzania	Refugees	n/a	Descriptive study	Used four methods—supervised classification, unsupervised classification, multi-resolution segmentation and mathematical morphology analysis—to identify refugee camps using IKONOS imagery.	Identified tents in refugee camps, subsequently managed to derive number of refugees and then created map of the camp. Found similar classification error rates (10 and 15%) as other methods, suggesting using this methodology for other geographical settings is applicable.
Hocini et al., 2018 [104]	Greater Mekong Subregion	MMPs	Malaria	Methods paper	Will use a focal test and treat intervention. Thirty peer navigators will be sent to seek out non-village based MMP. They will use GPS (global positioning system) to characterize movement patterns. They will collect data on time spent outdoors, outdoor movement during evening and dawn, time spent in forest, distance traveled and frequency of travel. Goal result is to examine collected GPS data and identify MMPs, MMPs with malaria, high density MMP areas and possible transmission hot spots.	Study ongoing.
Munoz et al., 2020 [95]	Venezuela	Migrants	n/a	Methods paper	Used Big Data to design a sampling frame to enumerate Venezuelan migrants in Ecuador. Employed Telefonica de Ecuador to implement three-phased sampling strategy: (1) Mobile phones were tagged as active, (2) Active phones were tagged as likely belonging to Venezuelans, (3) Active Venezuelan phones were assigned to primary sampling units where owner most likely resided.	The methodology is an adequate solution for enumerating migrants and identifying their location when censuses or central registries of migrants are not available.
Pelizari et al., 2018 [98]	Jordan	Migrants	n/a	Methods paper	Aimed identify built up settlements housing refugees. Approach embedded in object-based image analysis uses three components: (i) the computation of an exhaustive set of spectral-spatial features aggregated on multiple hierarchic segmentation scales, (ii) filter-based feature subset selection, and (iii) supervised classification using a Random Forest classifier.	Found all models had high accuracy values (ranging from 85.5 to 89.0%) of identifying built up settlements, temporary using multi-senor (MS and SAR) satellite imagery.
Research Innovation and Development for Health (RID4H), 2020 [72]	Burkina Faso	MMPs; IDPs	Trachoma; LF	Methods paper	An ongoing study that is identifying approaches for delivery of NTD surgical services to migrants and address barriers to receiving care. Methods include document review to assess facilities ability to provide MMDP services, use of mobile apps to track patients and refer care, key informant interviews (KIIs), focus group discussions (FGDs), and stakeholder buy-in.	Study ongoing.
Sightsavers, 2021 [101]	Cameroon	Nomadic pastoralists	NTDs; Onchocerciasis	Methods paper	Tested the ability of satellite imagery and GIS to remotely detect nomadic camps and help researchers target them for treatment. Obtained data through ESRI Geographical Information System software ArcGID PRO to produce and analyze spatial data. Then verified the camps in the field.	Field verification confirmed that more than 75% of camps identified as probable through satellite imagery were in fact camps. The imagery missed 8 camps known by local guides. Authors suggest this is fairly accurate but could be improved with high quality and recent satellite imagery.
Smith et al., 2019 [102]	Nepal	Migrant laborers	Malaria	Mixed methods	Used surveillance of passive and active imported case data and FGDs and KIIs to identify high-risk MMPs and areas where interventions could be adapted to target them. Modeled to investigate the association between indigenous case counts and importation rates. Findings suggest more than 50% of cases were imported. Most high-risk MMPs were adult migrant laborers. We are not able to coordinate surveillance when MMPs were leaving Nepal but were able to retrospectively survey population upon return from India.	Found that 54% of malaria cases reported between 2013 and 2016 were imported. There was a significant difference in gender such that male (85%) reported higher cases than females. Travel profiles suggest most MMPs travel to India for work for an average 3-day trip—increasing the risk of malaria transmission. In follow-up interviews, participants suggested most migrants would be willing and interested to participate in malaria screening if referred by a friend. Venue-based recruitment was also a well-liked option. Border screening was less popular.
Tompkins et al., 2016 [107]	Senegal	MMPs	Malaria	Methods paper	Analyzed Senegal mobile phone location data (from data 4 development) to determine characteristics of travel involving overnight stay which could impact malaria transmission. They defined “home” as place of most frequent calls and used 4 criteria to determine destination of travel. Then they calculated the proportion of trips involving an overnight stay. They created an agent-based model.	Found 60% of people have regular visits single destinations involving an overnight stay. Most visits involved a stay of only 1–2 nights. Findings suggest the ABM can approximately reproduce the patters of migration involving overnight stay. Authors note this study is limited as their reliance on mobile phone data may exclude those of lower socio-economic status.
Uzoma et al., 2019 [108]	Nigeria	Nomadic pastoralists	Polio	Methods paper	Mapped migratory routes of nomadic pastoralists in Borno State Nigeria. Their process included: stakeholder engagement, nomadic route mapping and validation, vaccination strategy for nomadic population, and tracking of nomadic vaccination activities (through GPS enabled smartphones). Central to their method was gaining community access through nomadic group leaders.	Successfully produced a map of nomadic routes using data from 4-step process. Found that nomads follow safe travel routes to avoid looting. The vaccination campaign was also successful—vaccinating nomadic temporary settlements or stops along the route. In 2017, 752 nomadic children received their first polio vaccination dose, which rose to 1155 in 2019 with this concerted nomadic vaccination effort.
Wangroongsarb et al., 2012 [110]	Cambodia, Myanmar, Thailand	Migrant laborers	Malaria	Mixed methods	Conducted a survey of migrant laborers in Thailand that came from Myanmar and Cambodia to determine demographics, migratory patterns, malaria knowledge and healthcare seeking tendencies. Employed respondent-driven sampling in lieu of cross-sectional or household survey methods due to lack of sampling frame. Trained health workers or survey staff in RDS and used coupons to recruit participants. Same size was approximately 1800.	Results suggest all migrants had come to Thailand due to working purposes. Healthcare utilization was higher among the Myanmar migrants compared to the Cambodian migrants (98 vs. 15%). The most predictive factor of treatment facility was proximity. The majority of the migrants had heard of malaria and knew it was transmitted by malaria (75–84%).
Wild et al., 2019 [111]	Ethiopia	Nomadic pastoralists	n/a	Methods paper	Developed a sampling strategy to survey mobile pastoralists by combining remote sensing and geospatial analysis. Used 0.5 m resolution satellite imagery of study area within 4 months of the survey. Implemented sampling frame using MCH indicators in Ethiopia.	Field validation confirms this method is comparable to conventional sampling frames. Authors suggest geospatial sampling methods used to enumerate mobile populations are cost-effective and logistically feasible.

An alternative sampling approach that has been successfully implemented is the engagement with MMPs themselves, such as participatory mapping and microplanning. This approach relies on local knowledge and local leaders or champions to identify barriers to access and points for intercepting target populations for services. In Nigeria, Uzoma and colleagues engaged with MMPs and completed route mapping to determine migratory routes and their potential contribution to polio transmission [108]. After successfully producing an accurate migratory map, a vaccination campaign was conducted which increased first dose vaccination coverage from 752 to 1155 nomadic children under 5 years of age over the 2-year campaign.

##### Intervention strategies to treat MMPs

We found 11 papers detailing methods used to implement mass health programming in MMPs (see Table 5) [8, 38, 73, 76, 80, 82, 88, 92, 94, 96, 100]. Five of these detailed approaches and successful engagement with members of the MMP as community health workers to provide ongoing service delivery, identify disease transmission hotspots, and engage in ongoing health education in a community [8, 88, 92, 94, 96]. For example, Hu et al. [88] implemented an expanded vaccine program in China—including more frequent services, provided by migrant clinical attendants; expanded social mobilization; and widened screening to identify migrant demands for vaccines—and observed an increase (71.5 to 88.6%) in fully vaccinated migrant children. These studies emphasize the need for altering common implementation approaches to include MMPs. Some of these include targeting interventions to specific known MMP groups within target districts, adjusting intervention coverage to include remote areas, and offering interventions at multiple sites (often along borders or at work sites with known migrant workers) and times to account for seasonal movement [8].

**Table 5.** Summary of intervention strategies used to target MMPs for treatment of focus diseases

Study	Geography	Target MMP group	Disease sampled or treatment method	Type of paper/study	Method	Results
Abakar et al., 2016 [76]	Africa	Nomadic pastoralist	Zoonotic diseases	Methods paper	Authors investigate OneHealth approaches to deliver services to MMPs. They reviewed various methods used to integrate surveillance systems between human and animal health to provide care to pastoralists.	Suggested collaboration between veterinary services and health services to reach pastoralists in remote areas could lead to more efficient implementation, higher coverage, and lower costs. Programs have integrated programming using community health and community animal health workers (CAHWs) simultaneously and coordinating with MOH and veterinary services at the national level to share transportation and equipment between programs. Authors suggest future programs should employ community-based syndromic surveillance for both human and veterinary disease. This alongside visual mobile phone technology can supplement existing health surveillance systems and improve the quality of surveillance for nomadic pastoralists.
Bechir et al., 2004 [80]	Chad	Nomadic pastoralist	Vaccine-preventable illnesses	Cross sectional	Aimed to increase vaccination coverage in Chad among nomadic pastoralists. Provided vaccination in conjunction with existing veterinary services, evaluated feasibility and limitations, determine what other services could be provided concurrently with veterinary services, and estimate cost saving.	Confirmed the feasibility of joint campaigns. Information, Education, and Communication efforts were adapted for nomadic pastoralists which proved effective. Conclude that by doing joint campaigns, vaccination can be provided to nomadic children and women in countries w/ limited resources.
Bomoi et al., 2016 [82]	Nigeria	Nomadic pastoralist	Polio	Methods paper	Describes an integrated human and animal vaccination strategy with the aim of increasing access and demand for routine immunization services among Nigerian Fulani nomadic pastoralists. Vaccination teams were comprised of local veterinary officers, healthcare workers, and health promotion officials.	Reported an increase in vaccination coverage from 22.7 to 80.1% in the sample of over 5000 children less than 1 year old and adult women. Animal vaccination coverage increased as well from 41 to 61%.
Hu et al., 2015 [88]	China	Migrants	Vaccine-preventable illnesses	Methods paper	Implemented an expanded program on immunization (EPI) and monitored the impacts on vaccination coverage, maternal understanding of vaccine program, and local immunization service performance among migrants in China. Intervention package included expended EPI service time and increasing frequency of vaccination service, training program for vaccinators, developing screening tool to identify vaccine demands among migrant clinic attendants and social mobilization for immunization. Obtained data from random sample investigation, vaccine service stats and qualitative interviews w/ vaccinator, and questionnaires with mothers of migrant children.	Immunization registration rate increased significantly, from 90.3 to 96.6% over the 32 months of implementation. The rate of fully vaccinated migrant children also rose as a result of the EPI from 71.5 to 88.6%.
Kheang, 2014 [92]	Greater Mekong Subregion (GMS)	MMPs	Malaria	Mixed methods	Established concern of malaria transmission among agricultural workers in GMS. Project implemented multi-pronged approach to provide malaria information and services to migrant workers that pass through while working or upon return home. Information is communicated by transportation services (taxi, buses) taking migrants to work, upon arrival or departure at malaria posts at boarder, or at malaria border clinics. Employed mobile malaria workers and mobile clinics at locals with high concentrations of migrants. Collaborated with large and small agriculture companies to supply LLINs during employment. Also used radio to disseminate malaria messaging.	Study ongoing.
Kleinschmidt et al., 2017 [73]	Southern Africa (SADC)		Malaria	Methods paper	Described program targeting malaria elimination in Southern Africa. Programs are establishing static and mobile border health facilities on 5 key international borders between high and low transmission districts. Goal is to improve access to malaria treatment for MMPs.	Study ongoing.
Ndiaye et al., 2014 [96]	Chad	Nomadic pastoralist	Polio	Methods paper	Polio eradication program implemented vaccination campaign in Chad. Vaccinated nomads in 2 regions by using mobile vaccination teams, recruiting local nomads, using social mobilization, and offering vaccinations to children, women, and animals.	Resulted in increased vaccination among nomads in intervention districts compared to control districts (e.g., increased 176% among nomadic children in intervention district compared to the control district which saw a decrease of 71% in vaccinations in nomadic children). Attributes success to (1) appointment of staff to oversee implementation, (2) engagement of the national government and its partners, (3) participation of nomadic community leaders, (4) intersectoral collaboration between human and animal health services, and (5) flexibility and capacity of vaccinators to vaccinate when and where nomads were available."
Hadarov et al., 2016 [100]	Somalia	Nomadic pastoralist	Polio	Descriptive study	Aimed to track nomadic pastoralist groups in Somalia and build trust with them to encourage polio vaccination adherence. Created a network of informants, engaged with clan leaders, mapped water points and livestock markets, formed partnership w/ animal vaccination efforts, collaborated across borders, established transit vaccination points.	Saw a reduction in zero-dose population due to the program (44.6% pre intervention and 19.5% post intervention). Noted increase cost with this type of intervention.
Shafique et al., 2011 [94]	Greater Mekong Subregion	MMPs	Malaria	Methods paper	This study piloted positive deviance, an “asset-based behavior change approach ... that suggests every community has certain individuals (positive deviants/champions) whose malaria prevention and treatment practices result in better health outcomes than their neighbors - in Cambodia.” The pilot aimed to identify and promote good health seeking practices. The intervention included FGD and interviews, highlighting positive behaviors which were then shared with the community to encourage others to do the same.	Preliminary results from the follow-up study suggested beneficial results from positive deviance such that it can serve as a malaria intervention targeting migrants for treatment.
The Global Fund, 2019 [8]	Global	MMPs	Malaria	Technical brief	Describes considerations taken when implementing mass treatment for malaria in migrant populations. Discusses various factors that complicate administering standard vector control measures to migrants such as gender, linguistic, culture, and ethnic barriers.	The brief describes including MMPs in decision-making regarding health policies and programming at the community level. Additionally, the brief details alternative methods of distribution (e.g., “adding additional distribution points, distributing door to door, using continuous distribution strategies rather than a mass campaign should be considered”) to increase coverage to MMPs.

Three articles highlighted One Health approaches that identify multiple entry points (such as veterinary care, agricultural extension, and other community points of entry) as a platform to engage the community in discrete health interventions [76, 80, 82]. For example, Bomoi et al. [82] reported successful use of joint animal and child vaccination, which improved childhood vaccination rates from 22.7 to 80.1% in Nigerian Fulani nomadic pastoralists over the course of their study; concurrently, animal vaccination rates also rose from 41 to 61%.

Lastly, two studies illustrate cross-border collaboration as another important strategy in engaging with MMPs [73, 100]. While the studies noted and described cross-border migration as a complicating factor that influences MMPs’ access to health care and disease risk (see outcome domains 1 and 2), Kleinschmidt et al. [73] and Haydarov et al. [100] suggest cross-border coordination as an approach towards lessening transmission risks and improving access to care. Means of cross-border collaboration ranged from formal efforts such as timing MDA on both sides of the border to coincide with one another, to informal WhatsApp groups that include communication between community health workers or other health service providers on both sides of the border. Informal communication channels have been used to communicate about movement, any increases in numbers of cases to assist in deploying rapid responses to community movement and/or increased morbidity.

A final observation that emerged under this outcome domain is the importance of planning and logistics. Emphasis is placed on the need to predict population movement and procure enough commodities, and providing services to MMPs will require greater resources—in time, human capital, and funding—than working with settled populations [22, 80, 97, 114].

## Discussion

To our awareness, this is the first attempt to systematically review the literature on motivation for and consequences of MMP movement on disease control and elimination progress at a large scale. A recent systematic review by Gammino et al. [6] did review the uptake of health services, including for NTDs, in nomadic populations; however, it did not include studies or data on the role of MMP movement, approaches to enumeration, or mass treatment and interventions.

We extensively reviewed the existing literature on MMP movement patterns, how MMPs contribute to the transmission of infectious diseases, and what tools and approaches have been tested to monitor MMP movement and target them with health interventions—this resulted in a robust library that describes the role of MMPs in the control and elimination of PC-NTDs. The review’s findings will be critical to inform operational research as NTD programs shift focus to systematically missed persons and other population groups that could be contributing to continuing transmission of disease [97]. Undertaking better NTD service provision for MMPs is a critical issue in health equity as many MMP groups have reduced access to healthcare, even if NTDs are also common among those living in poverty or with poor water, sanitation, and hygiene (WASH) infrastructure, conditions which some MMP groups are also at particular risk of enduring [8, 123].

### Implication for programmatic research

The reviewed literature suggests that designing a universal approach to target MMPs with NTD mass treatment—and more generally NTD interventions—is difficult. MMPs travel for different reasons, have different movement patterns, and may engage differently with their environment, leading to variable exposures to infection and disease. East Africa was the focus of this review, and the literature suggests there is high mobility between Uganda and Tanzania along the southeast and northwest borders. Additionally, the northeast border of Tanzania experiences high flow of MMPs with those coming from the southern border of Kenya [23]. Reviewed literature suggests population movement in East Africa, along with cultural practices, impacts healthcare utilization and access [17, 23, 46]. Therefore, to target MMPs for NTD interventions, it is crucial to understand the movement patterns, cultural practices, and healthcare acceptance among the specific populations.

Movement patterns and healthcare service utilization similarly affect how MMPs contribute to disease transmission. This review provides foundational information to the NTD community as programs investigate whether MMPs do in fact contribute to and could be the reason for persistent transmission of NTDs in certain geographic areas. The published literature on NTDs in MMPs suggests movement could possibly (re)establish foci of transmission in the destination upon arrival or in place of origin upon return if population movement is occurring in endemic areas [26, 27, 37, 38, 41, 43, 45, 47, 56]. Movement can also impact population’s knowledge about disease risk [15, 44]. The overwhelming consensus that population movement contributes to disease persistence is critical knowledge for NTD programs as they attempt to treat the most hard-to-reach populations.

While the literature confirms MMPs do impact persistent transmission of NTDs and that programs will need methods and approaches to monitor and treat these populations, no one single solution or approach to reach and provide interventions to MMPs is apparent. Therefore, programs will have to assess their context-specific situation, identify drivers of health care access, and choose the most applicable solution and approach. The literature suggests that the initial step of sampling MMPs can be the most difficult, as these groups are often not captured in central registries like population censuses [115]. Alternative programmatic options for sampling MMPs include using geospatial data, mobile phone records, respondent-driven sampling, and point intercept methods [72, 77, 86, 98, 101, 102, 107, 111]. Participatory mapping and micro-planning with the community themselves can also be an effective alternative to census-based sampling. This requires researchers to build trust with community leaders and gain entrance into the community to accurately sample them. It is important to note that MMPs believed to be contributing to the persistent transmission must be assessed in terms of their size, composition, and movement history within the context of a specific and larger socio-epidemiological context. Only then can barriers to access, whether physical, language, financial, or other be mitigated effectively.

This descriptive review also provides NTD programs with guidance for administering interventions once the population has been sampled. The literature illustrates three novel methods health programs have utilized which could become common practice for NTD programs if further refined and receiving appropriate financial support. Creating mobile teams by training MMP group members as migrant health workers; aligning with OneHealth principles by using platforms such as agriculture, veterinary extension, or WASH to also provide NTD-related activities; and improving cross-border coordination are approaches that have all been successfully piloted in the infectious disease sphere [38, 73, 80, 82, 92, 94, 100]. Employing these methods individually or in combination could support NTD programs in reducing persistent transmission in areas with known MMP contribution to residual NTD transmission.

As NTD programs plan concerted efforts to target MMPs for treatment, this review highlights important findings from other health sectors, as well as initial guidance for engaging with MMPs. The literature clearly establishes the consequential impact of untreated MMPs on persistent disease transmission. Implementation tools and strategies highlighted in this review can aid programs to improve implementation plans to better reach MMPs with treatment and improve disease surveillance.

### Policy considerations

While the results from this review are perhaps more applicable for operational research settings, there are some relevant program implications that policy makers and public health practitioners should consider. Based on the evidence reviewed, there are implications for commodity procurement, cross-border policies, and non-citizen healthcare access. First, the literature suggests that governments should consider allocating sufficient funds to procure commodities necessary to implement mass treatment programs in MMPs, regardless of distance and difficulty of reach. Sufficient financial and physical resource procurement is vital to successfully treating last mile populations contributing to NTD transmission. Second, studies and experts in the field encourage national health ministries to engage with neighboring health ministries to detail plans for cross-border engagement. This has been shown to improve programming in MMPs, including aligning the timing of health education and MDA campaigns. Third, the review highlights the epidemiologic importance of treating everyone regardless of legal or citizenship status. Treating everyone is not only critical from a human rights perspective but also vital to the control and elimination of diseases, as MMP movement—just like etiological agents, their vectors, and/or reservoirs—is incognizant of national boundaries.

### Study limitations

There are limitations to the review related to both the evidence pulled from the search and the analysis. We purposefully confined the search to actively mobile persons. Therefore, we intentionally excluded additional literature on refugees in camps or other semi-settled populations. Literature on refugees is quite vast and could have provided additional contextual information on knowledge, attitudes, and practices in that particular MMP group. We also limited the search geographically. East Africa was the focus of the review, and we searched the literature on specific populations such as the Maasai and Turkana. We acknowledge that these better-known groups are more likely to be the subject of research, possibly yielding an over representation of these groups in our review of the literature. Future research should focus on MMPs in other geographic regions in Africa and elsewhere. It is for this reason that we do not assert that the literature we found is exhaustive in addressing all MMP populations.

The analysis of the studies itself was limited. The review was qualitative in nature; therefore, quantitative analyses of the efficacy of sampling methods or of the reliability of prevalence results were not conducted. While the findings are critical to inform program planning, subsequent analyses will need to be conducted to address the validity of the methods proposed in this review. Furthermore, the inevitability of human error limited our refinement process of the review. While the inclusion and exclusion criteria were explicit and we engaged a review team of three independent reviewers, there is still chance for difference in subjective interpretation.

We also acknowledge that some studies used convenience or snowball samples which can lead to bias. Therefore, while our analysis captures lessons from extant literature, we cannot be sure that the studies are wholly representative of the populations they are describing.

## Conclusions

In summary, we identify many sampling and intervention approaches that have been used to improve health outcomes for MMPs across reviewed focus diseases. These approaches, where successful, have been predicated on a good understanding of the MMPs themselves, their demographics, movement patterns, and the epidemiological risk factors they experience in their places of origin and in destination geographies. In almost every case, successful strategies we identified were rooted in active effort to seek these populations out and to meet them where they are, actively lowering the barriers to prevention, diagnosis, and treatment services. Actively engaging these populations is critical, firstly, from a health and human rights perspective (as these groups are so often vulnerable or otherwise marginalized). However, working with, as opposed to around, MMP groups is also a socio-epidemiological imperative in some geographies, if the global health community is going to stop transmission and achieve disease control and more importantly elimination goals.

## Data Availability

Data sharing is not applicable to this article as no datasets were generated or analyzed during the current study.

## Abbreviations

ASTMH: American Society of Tropical Medicine and Hygiene
BMGF: Bill and Melinda Gates Foundation
CDC: Center for Disease Control and Elimination
CAHW: Community animal health worker
COR-NTD: Coalition for Operational Research on NTDs
EPI: Expanded Program on Immunization
FGD: Focus group discussion
GMS: Greater Mekong Subregion
GPS: Global positioning system
HPM: Human population movement
IDP: Internally displaced person
ITI: International Trachoma Initiative
KAP: Knowledge, attitude, practice
KII: Key informant interview
LLINs: Long lasting insecticidal nets
LF: Lymphatic filariasis
MDA: Mass drug administration
MMDP: Managing morbidity and disability prevention
MMP: Mobile and migrant population
NTD: Neglected tropical disease
NNN: NTD Non-Governmental Development Organization Network
PC: Preventive chemotherapy
PICO: Problem/population, intervention, comparison, outcome
PROSPERO: Prospective Registry of Systematic Reviews
PRISMA: Preferred Reporting Items for Systematic Reviews and Meta-Analyses
RTI: Research Triangle Institute
SCH: Schistosomiasis
STH: Soil-transmitted helminths
TCC: The Carter Center
WASH: Water, sanitation, and hygiene
WHO: World Health Organization
